# Towards Universal Health Coverage: An Evaluation of Rwanda *Mutuelles* in Its First Eight Years

**DOI:** 10.1371/journal.pone.0039282

**Published:** 2012-06-18

**Authors:** Chunling Lu, Brian Chin, Jiwon Lee Lewandowski, Paulin Basinga, Lisa R. Hirschhorn, Kenneth Hill, Megan Murray, Agnes Binagwaho

**Affiliations:** 1 Department of Global Health and Social Medicine, Harvard Medical School, Boston, Massachusetts, United States of America; 2 South Asia Department, Asian Development Bank, Metro Manila, Philippines; 3 Department of Community Health, National University of Rwanda School of Public Health, Kigali, Rwanda; 4 Department of Global Health and Population, Harvard School of Public Health, Boston, Massachusetts, United States of America; 5 Department of Epidemiology, Harvard School of Public Health, Boston, Massachusetts, United States of America; 6 Ministry of Health, Government of Rwanda, Kigali, Rwanda; Groningen Research Institute of Pharmacy, United States of America

## Abstract

**Background:**

*Mutuelles* is a community-based health insurance program, established since 1999 by the Government of Rwanda as a key component of the national health strategy on providing universal health care. The objective of the study was to evaluate the impact of *Mutuelles* on achieving universal coverage of medical services and financial risk protection in its first eight years of implementation.

**Methods and Findings:**

We conducted a quantitative impact evaluation of *Mutuelles* between 2000 and 2008 using nationally-representative surveys. At the national and provincial levels, we traced the evolution of *Mutuelles* coverage and its impact on child and maternal care coverage from 2000 to 2008, as well as household catastrophic health payments from 2000 to 2006. At the individual level, we investigated the impact of *Mutuelles*' coverage on enrollees' medical care utilization using logistic regression. We focused on three target populations: the general population, under-five children, and women with delivery. At the household level, we used logistic regression to study the relationship between *Mutuelles* coverage and the probability of incurring catastrophic health spending. The main limitation was that due to insufficient data, we are not able to study the impact of Mutuelles on health outcomes, such as child and maternal mortalities, directly.

The findings show that *Mutuelles* improved medical care utilization and protected households from catastrophic health spending. Among *Mutuelles* enrollees, those in the poorest expenditure quintile had a significantly lower rate of utilization and higher rate of catastrophic health spending. The findings are robust to various estimation methods and datasets.

**Conclusions:**

Rwanda's experience suggests that community-based health insurance schemes can be effective tools for achieving universal health coverage even in the poorest settings. We suggest a future study on how eliminating *Mutuelles* copayments for the poorest will improve their healthcare utilization, lower their catastrophic health spending, and affect the finances of health care providers.

## Introduction

*Mutuelles de santé* (*Mutuelles*) is a community-based health insurance program established by the Government of Rwanda (GoR) as a key component of the national health strategy on providing universal health care and reaching the health Millennium Development Goals (MDGs). Recent years have witnessed a global re-emergence of support for achieving universal health care [1]. Two major goals of universal coverage have been clearly outlined: to ensure access to care for those in need, and to provide financial risk protection by lowering catastrophic out-of-pocket health spending. Existing studies have shown that catastrophic health spending pushes households into poverty in both developed and developing countries [2]–[6]. Insuring underserved populations has been considered a useful means of improving access to care with financial risk protection. The existing evidence shows that in countries such as Mexico, China, Vietnam, Ghana, and Mali, government-sponsored or community-based insurance programs for uninsured populations improved access to curative care [7]–[13]. However, the results of studies on financial risk protection vary widely - the programs had little or no impact in Vietnam and China, [9]–[11] while Mexico's program had a significant effect in reducing household catastrophic health spending [7], [8]. In Mali and Ghana, the programs resulted in protection against potentially catastrophic expenditures related to hospitalization, but did not appear to have a significant effect on out-of-pocket expenditures for curative outpatient care [13].

This paper presents a case study on Rwanda, a small country in central east Africa with a population of 10 million in 2009 [14]. After the genocide in 1994, Rwanda has been making impressive progress in its social and economic development. The GDP per capita increased from 240 USD (constant 2009 USD) in 2001 to 510 USD in 2009 [14]. Nevertheless, Rwanda remains one of the poorest countries in the world, with about 57% of its population living below the national poverty line (0.45 USD per adult per day) and 37 percent living in extreme poverty (0.32 USD per adult per day) [15].

Before 1999, the majority of the population in Rwanda had no health insurance. The uninsured population had to pay for health services out-of-pocket. Facing limited resources, the GoR has been implementing *Mutuelles* since 1999 to provide affordable basic services, especially child and maternal care, to the uninsured population. A pilot program was implemented in three selected districts in 1999 and 2000. The success of the pilots motivated the local governments and communities to quickly adopt and expand the program nationwide. To standardize the main parameters of *Mutuelles*, such as the benefits package, enrollment fees, subsidization mechanisms, organizational structure, management systems, etc., the *Mutuelles* Health Insurance Policy was approved by the GoR at the end of 2004. Until it was fully implemented in 2006, there was variation and flexibility in scheme design across districts. In 2008, a law on the creation, organization, and management of *Mutuelles* was enacted, which further strengthened the strategy [16].

Approximately 50 percent of *Mutuelles'* funding is comprised of annual member premiums. The remaining half is obtained via transfers from other insurance funds, charitable organizations, NGOs, development partners, and the GoR. Providers are paid by *Mutuelles* directly, either through monthly capitation rates on a fee-for-service basis, or via (recently introduced) performance-based payments [17].

*Mutuelles* uses a policy of household subscription. Before 2007, the annual premium for a household with up to seven members varied across regions, ranging typically from 2,500 to 11,500 RWF (4.72 to 20.83 current USD). Since 2007, the annual premium has been 1,000 RWF (1.81 current USD) per member [16]. With the support from donors such as the Global Fund to Fight AIDS, Tuberculosis and Malaria, the enrollment fees for the poorest 16^th^ percent of the population is exempt [18].

Enrolled households are affiliated to designated health centers. With referrals from the health center, members may obtain hospital services covered by *Mutuelles*. To mitigate adverse selection, enrollees must wait one month to utilize covered services. Before 2006, *Mutuelles* covered all services and drugs in the health center and limited services (such as C-sections and related hospitalization) in the hospital. After 2006, *Mutuelles* enrollees were entitled by law to a minimum service package (PMA) at the health center and a complementary service package (PCA) at the district hospital described in Table 1. In practice, the MoH estimates that only 30 percent of health centers provide the comprehensive list of activities [17].

**Table 1:** Services provided at health centers and district hospitals covered by *Mutuelles.*

Facilities	Service Provided	Contents of the Service
Health centers	Minimum Package of Activities (PMA)	***Promotional activities***
		Child growth monitoring, community-based health insurance, psychosocial support, community involvement, home visits, information, education and communication for health
		***Preventive activities***
		Vaccination, prenuptial consultations, prenatal and postnatal care, voluntary consultation and testing for HIV, family planning, water and sanitation, school health services and epidemiological monitoring
		***Curative activities***
		Curative consultations, child health care, management of chronic illnesses, nutritional rehabilitation, HIV/AIDS patient treatment, curative care, normal deliveries, minor surgery and laboratory tests, drug provision
District hospitals	Complementary Package of Activities (PCA)	Prevention, including preventive consultations for referred cases and prenatal consultations for at-risk pregnancies; family planning, with all methods available for those referred, including tubal ligation and vasectomy; curative case for those referred, including the management of difficult and caesarean deliveries, medical and surgical emergencies, minor and major surgery, hospital care, drug provision, laboratory analyses and medical imaging; and management, including training for paramedical staff and supervision

Source: Ministry of Health, Rwanda.

Before 2006, copayments per visit to health center typically varied from 100 to 150 RWF (0.30 to 0.45 current USD) and cost up to 50% of the hospital fee. After 2006, copayments for a health center visit have been 200 RWF (0.36 current USD) and 10% of the hospital fee for hospital services [16].

To date, Rwanda is the only country in sub-Saharan Africa where more than 90% of the population is covered by community-based health programs [19]. To enhance cross-country learning and gather evidence for future policy-making in Rwanda, we conducted an empirical evaluation of *Mutuelles'* impact on universal health coverage. Existing empirical evaluation of *Mutuelles*' impact on universal health coverage is limited and subject to various issues. Most of them focused only on the relationship between *Mutuelles* enrollment and medical care utilization, and were based primarily on the data collected in the three pilot districts in 2000 [20], [21]. One study used the Rwanda Demographic and Health Survey (RDHS) in 2005 to examine the effect of *Mutuelles* on medical care utilization; however, it did not address adverse selection, an issue which could lead to inconsistent estimates of *Mutuelles*' impact [22]. Among all existing studies, only one examined the impact of *Mutuelles* on universal financial risk protection using the Integrated Living Conditions Survey (EICV) [23]. The study was limited to the year 2005/2006 and did not trace the change in catastrophic health spending after the establishment of *Mutuelles*. When calculating household out-of-pocket health spending, the study did not include household payments on vaccination or transportation to health facilities. The EICV 2005/2006 collected household spending on medical services with a 12-month recall period and a 2-week recall period. The study used a 2-week measure for spending on outpatient services and 12-month measure for spending on inpatient services. No explanation was given as to why the measures were chosen. A previous publication on 43 developing countries showed that estimates of spending on inpatient services are very sensitive to the choice of recall period [24]. In addition, the study did not make the effort to deal with endogenous household expenditure, an important confounder included in the study. Issues aforementioned raise concerns of the consistency and accuracy of the estimates generated from these studies.

Using two nationally and geographically representative population surveys: (1) the Integrated Living Conditions Survey in 2000 and 2005/2006; and (2) the Rwanda Demographic Health Survey in 2000, 2005, and 2007/2008, our paper provides the first systematic quantitative analysis of *Mutuelles'* impact on universal health coverage in its first eight years of implementation. Methodological issues that hampered previous *Mutuelles* studies, such as selection bias in utilization analyses, estimating household out-of-pocket health spending, and endogenous household expenditure in financial risk protection, have been addressed with various statistical methods.

## Methods

Our study takes a comprehensive approach and is executed at multiple levels over an 8-year period. We traced the temporal trends of child care, maternal care, average annual household out-of-pocket health spending, percentage of households with catastrophic health spending, and *Mutuelles* enrollment at the national level; we ascertained the relationship between child/maternal care coverage and *Mutuelles* coverage at the provincial level; and we examined the impact of *Mutuelles* enrollment on financial risk protection at the household level and on medical care utilization at the individual level. For the individual utilization analysis, we focused on three populations: the general population; under-five children with diarrhea, fever, or acute respiratory infection (ARI); and women who gave birth in the survey years.

### Data Sources

The Integrated Living Conditions Survey and the Rwanda Demographic Health Survey are the only two household surveys conducted at the national level in Rwanda. They have been used frequently for providing national and regional evidence to policy makers in the country. Both surveys are cross-sectional. Households included in the two surveys are selected from the same sample cells. The EICV collects data every five years on household expenditures, consumption, demographic and socioeconomic characteristics, information on health insurance status, self-reported illness, medical care utilization, self-reported out-of-pocket health spending on medical services, etc. The survey is conducted over a 12-month period to address seasonality issues. The available data includes the EICV from 2000 and 2005/2006. Seventy-five percent of the households were interviewed in 2006, and we will refer to the survey as EICV 2006.

The RDHS collects information from women on child health and care, maternal health and care, socio-demographic indicators, health insurance, and a number of other health indicators. Since more than 90 percent of the interviews were conducted in 2008 for the RDHS 2007/2008 survey, we will refer to it as RDHS 2008. To increase the sample size for the child and maternal care analyses, we pooled RDHS data from 2005 and 2008 and used them in the regression analyses. Tables 2 and 3 present the total sample size of the surveys and the number of individuals and households included in our analyses.

**Table 2:** Households and individuals included in the analyses of financial risk protection and medical care utilization with EICV.

	EICV 2000	EICV 2006
Total number of individuals	32,153	34,785
Number of individuals included in medical care analyses	8,209	6,334
Total number of households	6,420	6,900
Number of households included in financial risk protection analyses	6,408	6,280

**Table 3:** Individuals included in the analyses of child and maternal care with RDHS.

	RDHS 2000	RDHS 2005	RDHS 2008
Total number of interviewed women	10,421	11,321	7,377
Number of women included in maternal care analyses	1,290	764	1,091
Total number of under-five children	7,033	7,797	5,489
Number of under-five children included in child care analyses	2,671	2,796	1,837

The sampling method and questionnaires of RDHS are standardized over time, enabling the construction of time-series data at the provincial level for analyzing the relationship between *Mutuelles* coverage and child/maternal care coverage. Before 2006, there were 12 provinces in Rwanda. In 2006, the 12 provinces were reorganized into five regions. The RDHS 2008 includes a variable indicating the previous provinces, which allowed us to generate information for the 12 provinces in 2008. We excluded Kigali city and its surrounding rural areas since the population in those areas have a different socioeconomic profile from populations in other provinces. Ten provinces were included in the provincial-level study. Each panel had a total of 30 observations over 2000, 2005, and 2008.

### Study Samples and Variables

To study the impact of *Mutuelles* on universal health coverage, we included in our analysis only individuals and households that were either without any health insurance or were covered only by *Mutuelles*. We excluded those with other health insurance plans. We studied the impact of *Mutuelles* on protecting households from financial risk among *Mutuelles*-insured households and uninsured households. For medical care utilization, we restricted the regression analyses to those who reported being sick in the two weeks prior to the surveys for the general population, under-five children, and to women with deliveries in the survey years. We did not include the utilization of preventive care, such as vaccination for children. Free vaccination has been provided to all children in Rwanda regardless their health insurance status. Together with a strong community health network and media education, free vaccination has contributed to the high rate of vaccination coverage in Rwanda. For example, in 2005, about 97 percent of children age 12–23 months received BCG vaccine (*Baccille Calmette Guérin vaccine*) and about 95 percent of children received DTP3 vaccine (diphtheria, tetanus and pertussis vaccine) [25]. The high percentage of immunization coverage demonstrates little variation in preventive care utilization.

#### (1) Variables for analyzing the impact of Mutuelles on individual medical care utilization among general population (EICV 2006)

To investigate how enrolling in the *Mutuelles* insurance program influenced an individual's utilization of medical care when they were ill, we constructed an outcome variable indicating an individual using medical services when he or she was ill in the previous two weeks of the survey. Medical services included inpatient care, outpatient consultation, and medical tests and exams. In 2006, about 31.6 percent of individuals who reported an illness in the previous two weeks of the survey used medical care. We excluded those who had other types of insurance (6.4 percent) and kept 6,334 individuals in the study.

A dummy variable “*Mutuelles* coverage” was created to represent participation in *Mutuelles*. Socio-demographic variables included age, gender, household size, rural residence, schooling of the household head, and household expenditure quintiles. Dummy variables “no schooling”, “primary school or less” and “higher than primary school” referred to household heads with no schooling, less than or equal to primary schooling, or above primary schooling, respectively. “No schooling” served as the reference group in the analysis. Five dummy variables indicated household expenditure quintiles where quintile five (the highest expenditure) was the reference group. Two dummy variables “time to health center” and “time to hospital” indicated travel time of more than 1 hour to the nearest health center and more than 2 hours to the nearest hospital. “Radio ownership” was created to measure the effect of public health education, which is usually conducted through radio programs in Rwanda.

A dummy variable “severity of the illness” was constructed, indicating whether or not an individual who self-reported illness had to stay in bed due to the severity of the illness. Another dummy variable “disability” indicated whether or not a person suffered from any kinds of disabilities at the time of survey. To control for heterogeneity of health systems-related variables across districts, we constructed district dummy variables and included them in the regression analyses.

#### (2) Variables for analyzing the impact of Mutuelles on individual medical care utilization among under-five children and women with delivery (pooled RDHS 2005 and 2008)

The outcome variable “childcare” indicates whether a child under-five received medical care when having acute respiratory illness (ARI), fever, or diarrhea. For a woman who delivered a child in the survey year, we constructed an outcome variable indicating whether or not she had skilled-birth attendance during the delivery.

Independent variables included *Mutuelles* coverage, age and gender of the household head, age and schooling level of the child's mother (0 = no schooling, 1 = otherwise), wealth quintiles, rural residence, and radio ownership. A year indicator was constructed (1 = year 2008, 0 = year 2005) to address unobserved confounders that may vary between the two years.

#### (3) Variables for analyzing the effect of Mutuelles on financial risk protection (EICV 2006)

Using EICV 2006, an outcome variable was constructed to study the impact of *Mutuelles* on protecting households from financial risk: a dummy variable indicating a household with catastrophic health spending. We used the definition of catastrophic health spending proposed by the World Health Organization: a household has catastrophic health spending if its annual out-of-pocket health expenditure exceeds 40 percent of annual capacity to pay, where capacity to pay is measured by household expenditure excluding spending on basic subsistence needs. The basic subsistence needs is calculated as the average annual food expenditure of households whose food share is in 45^th^ and 55^th^ percentile [3].

A household's annual out-of-pocket health payment includes its spending on medical care and travel to health facilities. The data for outpatient and inpatient care, medicine, lab tests, and transportation were collected for a recall period of two weeks in the EICV 2000 and 2006, and a recall period of 12 months in the EICV 2006. It has been found that the choice of recall period may significantly affect the measurement of household health spending [24]. To ensure comparability of the estimates between 2000 and 2006, we used 2-week measures for household health spending on outpatient and inpatient care, medicine, lab tests, and transportation to health facilities. We derived annual estimates for these items by timing 26. Spending on vaccinations in the last 12 months was also included in the total household out-of-pocket health spending.

Independent variables included *Mutuelles* coverage, age, gender, and schooling of the household head, household size, rural residence, expenditure quintiles, and two dummy variables “time to health center” (1 hour) and “time to hospital” (2 hours). We also constructed dummy variables for households with under-five children, the elderly (age over 60), and household members with disability, since these variables may have been related to the needs of medical care.

Household economic status was measured by household total expenditure. The items included in the total expenditure calculation were education spending, housing spending, health spending, food spending (including self-made products and excluding alcohol, cigarettes, restaurants), spending on durable goods, agriculture, and other items.

We used the total expenditure of a household to measure its economic status. This rendered the expenditure variables endogenous since health spending was included in the calculation of household total expenditure. We dealt with this issue by using the housing area per household member as an instrument for household total spending. The housing area will not be a valid instrument if houses are sold to finance health care. Households in Rwanda rarely sell their homes. To check whether housing area was an effective instrument for household total expenditure, we conducted an F-test to determine whether the R-squared value from an unrestricted regression on household total spending (including housing area per capita as the instrumental variable) was significantly higher than that from a restricted regression (excluding housing area) suggested by Staiger and Stock [26]. The F-test value was 115 with a p-value of 0.001, indicating that housing area per capita is not a weak instrument. The correlation between the instrumented expenditure and expenditure variables was 0.70.

#### (4) Variables used in provincial-level analysis

For provincial level analyses, we constructed two outcome variables: “maternal care coverage” (skilled-birth attendance) and “child care coverage”, which measure the percent of target populations that obtain medical care when in need. The percentage of the population enrolled in *Mutuelles* was an independent variable. Other possible confounders were percentage of children's mothers/women with some schooling (versus no schooling), percentage of the studied population in the poorest wealth quintile, and time-invariant provincial fixed effects.

### Statistical Analyses

#### (1) Multiple level analyses

At the national level, we tracked the trends of *Mutuelles* coverage, average annual household out-of-pocket health spending, percentage of households with catastrophic health spending, under-five child care and skilled-birth attendance coverage, and child and maternal mortalities between 2000 and 2008. We also presented the likelihood of using medical care and incurring catastrophic health spending for both *Mutuelles* enrollees and uninsured populations across household expenditure quintiles after controlling for possible confounders.

At the provincial level, we used random-effects models (based on the Hausman test) with Huber-White robust standard errors to examine the relationship between *Mutuelles* coverage and child/maternal care coverage.

At the household level, we used logistic regression to estimate the impact of *Mutuelles* on the likelihood of a household incurring catastrophic health spending.

At the individual level, we used logistic regression models to estimate the impact of *Mutuelles* on medical care utilization among the three target populations when ill.

#### (2) Addressing selection bias in utilization analyses

Selection bias is a major concern when analyzing the impact of *Mutuelles* on medical care utilization: households may self-select into the *Mutuelles* due to observable or unobservable characteristics that may be correlated with medical care utilization. For example, households with members who are in poorer health are more likely to join the program, and they may use more medical care, holding all other things equal. The existence of selection bias may lead to an over-estimated impact of *Mutuelles* on individual medical care utilization.

To address the issue, we first examined whether *Mutuelles* enrollees were more likely to be sick or need care than the uninsured population. Table 4 shows that for the general population, about 17.8 percent of *Mutuelles* enrollees (95% confidence intervals between 17.1% and 18.5%) reported illness in the two weeks prior to the survey. Among the uninsured population, about 20.9 percent of individuals (95% confidence intervals between 20.3% and 21.5%) reported an illness, which was significantly higher than the *Mutuelles* enrollees. For under-five children and women, there was no significant difference in reported illness and delivery between uninsured individuals and *Mutuelles* enrollees. This suggests that *Mutuelles* enrollees were not more likely to have an illness or need care than the uninsured individuals.

**Table 4:** Checking endogeneity of *Mutuelles*: mean difference of self-reported illness and birth delivery by *Mutuelles* status (RDHS).

	Self-reported illness
	Mean (95% CI)
**General population 2006 (EICV 2006)**	
No insurance	0.209 (0.203, 0.215)
With *Mutuelles*	0.178 (0.171, 0.185)
**Under-five children 2005 (RDHS 2005)**	
No insurance	0.344 (0.330, 0.358)
With *Mutuelles*	0.314 (0.299, 0.330)
**Under-five children 2008 (RDHS 2008)**	
No insurance	0.348 (0.325, 0.370)
With *Mutuelles*	0.336 (0.319, 0.353)

	Self-reported delivery
**Women 2005 (RDHS 2005)**	
No insurance	0.063 (0.057, 0.069)
With *Mutuelles*	0.077 (0.068, 0.085)
**Women 2008 (RDHS 2008)**	
No insurance	0.154 (0.139, 0.170)
With *Mutuelles*	0.151 (0.140, 0.162)

We then examined the existence of selection bias due to observable characteristics by investigating the determinants of joining the *Mutuelles* program. Table 5 presents the logit regression results on the likelihood of a household participating in *Mutuelles* using EICV 2006. We found the following significant predictors of participating in *Mutuelles*: households in rural areas, heads of household with more than primary schooling, household size, radio ownership, and time to the nearest health center. Compared to households in the lowest expenditure quintile, households in the 3^rd^ and higher expenditure quintiles were more likely to join *Mutuelles*.

**Table 5:** Checking endogeneity of *Mutuelles*: logit regression results for household affiliation to *Mutuelles* (N = 6,381).

	Coefficient	SE	P Value
Rural residence	0.599	0.110	0.000**
Head: age 30–50	0.094	0.073	0.198
Head: age >50	0.229	0.120	0.056
Head: female	−0.115	0.069	0.094
Head: < = primary schooling	0.069	0.070	0.322
Head: >primary schooling	0.404	0.079	0.000**
Household size	0.377	0.055	0.000**
Expenditure quintile2	0.152	0.094	0.105
Expenditure quintile3	0.513	0.093	0.000**
Expenditure quintile4	0.686	0.094	0.000**
Expenditure quintile5	0.842	0.104	0.000**
Under-five children	−0.084	0.069	0.223
Elderly (≥60)	−0.088	0.117	0.455
Disability	−0.014	0.076	0.852
Radio ownership	0.410	0.058	0.000**
Time to health center (>1 hour)	−0.160	0.059	0.007**
Time to hospital (>2 hours)	0.037	0.066	0.577
Constant	−3.134	0.215	0.000**
Regional dummies (coefficient omitted)			

Abbreviations: SE: standard error.

* : statistically significant at the 0.05 level.

** : statistically significant at the 0.01 level.

To mitigate possible selection bias due to observable household characteristics, we constructed matched datasets with the propensity score matching (PSM) method to ensure that the observed characteristics of the control (uninsured population) and treatment (*Mutuelles* enrollees) groups were as similar as possible after being matched [27], [28]. The closeness of the two groups was measured by the difference in means of observable variables for the two groups. If the means of these variables were not statistically different from each other, the two groups were close enough to be matched. Following previous studies [9], [29] we used kernel matching that allows for more than one comparison unit to be matched with one treatment unit.

For utilization analysis among the general population with the EICV 2006 data, the matching variables included age, gender of the individual, schooling level of the household head, rural residence, household size, expenditure quintiles, travel time to the nearest health center and hospital, radio ownership, severity of the reported illness, and individuals with disability. The unmatched data included 6,334 individuals who reported illness in the prior two weeks, and the matched dataset included 5,435 individuals reported illnesses.

For utilization analysis among under-five children with ARI, fever, and diarrhea, with pooled data from the RDHS 2005 and 2008, matching variables included age and gender of the household head, mother's age and schooling, rural residence, wealth quintiles, and radio ownership. The unmatched datsaset included 4,633 under-five children who reported an illness. The matched dataset included 4,421 children under-five who had illness.

For utilization analysis among women with child delivery in 2005 and 2008, the matching variables included age and gender of the household head, women's age and schooling level, rural residence, wealth quintiles, and radio ownership. The unmatched dataset included 1,855 women who had delivery in the survey years. The matched dataset included 1,766 women who had delivery in the survey years.

The mean differences in matched variables between the uninsured population and *Mutuelles* enrollees for the three populations are presented in the Supporting Information section (Tables S1, S2, S3, and S4). They were not statistically significant at the 0.05 level, indicating that selection bias from the observed characteristics was substantially reduced in the matched data.

We examined the existence of selection bias due to unobserved factors using a two-stage residual inclusion (2SRI) method recommended by Terza et al [30]. In the first stage, we ran a logit regression on *Mutuelles* affiliation and obtained the residuals. In addition to random errors, the residuals may represent unobserved factors affecting household decisions of joining the program. In the second stage, we ran a logit regression on medical care utilization and included the residuals as a predictor. If the coefficient of the residuals is statistically significant, that indicates the existence of unobserved factors that are both correlated with *Mutuelles*' enrollment and medical care utilization. With the exception of under-five children, the coefficients of the residuals were not statistically significant at the 0.05 level (Table 6), indicating that we may not reject the null hypothesis of exogenous *Mutuelles* variable when conducting analyses for the general population, and women with delivery. To check the sensitivity of the findings, we produced a set of regression results for medical care utilization among the three populations using the Instrumental Variables (IV) method.

**Table 6:** Testing endogeneity of *Mutuelles* with two-stage residual inclusion method.

	Coefficients of residuals	P value	95% CI
General population (EICV 2006)	0.037	0.192	(−0.019, 0.093)
Under-five children (pooled RDHS 2005 and 2008)	−0.299	0.003**	(−0.493, −0.105)
Women with delivery (pooled 2005 and 2008)	−0.137	0.153	(−0.437, 0.163)

Abbreviations: CI: confidence interval.

* : statistically significant at the 0.05 level.

** : statistically significant at the 0.01 level.

An ideal instrumental variable should be closely correlated to a household's participation in the *Mutuelles* program, but has no relationship with the decision to use medical care, conditional on other covariates. Local governments played an important role in establishing and promoting *Mutuelles*, and a household's decision to participate in *Mutuelles* was affected by public campaigns. We constructed a measure of “cluster insurance rate” for each observed household: the average rate of *Mutuelles* enrollment by cluster, using all of the household's *Mutuelles* status information in the cluster other than the insurance for the observed household. In the first stage, we included this variable in the logit regression to analyze the determinants of *Mutuelles*' affiliation and obtained the predicted probability of participating in *Mutuelles* for each household. In the second stage (medical care utilization analysis), we replaced the *Mutuelles* coverage variable with the predicted probability of participating in *Mutuelles*. In this way, we obtained the impact of *Mutuelles* on outcome variables with the IV method. We checked whether or not the “cluster insurance rate” was a weak instrument. Take analysis of medical care utilization among the general population using the EICV 2006 as an example. In the first stage, the coefficient of the instrument was positive (2.802) and significant at the 0.001 level. This suggests that the instrument was directly related to a household's enrollment in *Mutuelles*. A likelihood ratio test was used to evaluate the difference between the nested models. The chi-squared value (377.93) was statistically significant at the 0.001 level, suggesting that the instrument was not weak in predicting a household's likelihood of participating in *Mutuelles*. We repeated the same procedure for under-five children and women with deliveries. We present regression results for the three target populations generated from the unmatched data, matched data, and matched data with IV method.

To be consistent with the utilization analyses, we also checked the sensitivity of the regression results for catastrophic health spending analysis with matched data and IV methods.

## Results

### Summary statistics of variables

Tables 7, 8, 9, and 10 provide summary statistics for variables used in individual utilization analyses (for general population, women with delivery, and under-five children) and household financial risk protection analyses. Information is provided for both the unmatched and matched data that excluded outliers after matching.

**Table 7:** Descriptive statistics for variables used in analyzing medical care utilization of the general population who reported illness in the prior two weeks of the survey (EICV 2006).

	Unmatched Data			Matched Data		
	N	Mean	SD	N	Mean	SD
***Dependent Variable***						
Utilization	6,334	0.300	0.458	5,435	0.305	0.461
***Independent variables***						
*Mutuelles* coverage	6,334	0.360	0.480	5,435	0.360	0.480
Age	6,334	24.32	21.24	5,435	24.11	21.14
Female	6,334	0.561	0.496	5,435	0.560	0.496
Head schooling: none	6,332	0.316	0.465	5,435	0.307	0.461
Head schooling: < = primary school	6,332	0.399	0.490	5,435	0.417	0.493
Head schooling: >primary school	6,332	0.284	0.451	5,435	0.276	0.447
Rural residence	6,334	0.787	0.409	5,435	0.816	0.388
Household size	6,332	2.583	0.627	5,435	2.583	0.617
Expenditure quintile1	6,332	0.190	0.391	5,435	0.167	0.373
Expenditure quintile2	6,332	0.199	0.399	5,435	0.205	0.404
Expenditure quintile3	6,332	0.199	0.399	5,435	0.217	0.412
Expenditure quintile4	6,332	0.206	0.405	5,435	0.209	0.407
Expenditure quintile5	6,332	0.207	0.405	5,435	0.202	0.402
Severity of illness	6,323	0.722	0.448	5,435	0.724	0.447
Disability	6,334	0.062	0.242	5,435	0.060	0.236
Time to health center (>1 hour)	6,334	0.385	0.487	5,435	0.375	0.484
Time to hospital (>2 hours)	6,330	0.636	0.481	5,435	0.637	0.481
Radio ownership	6,332	0.480	0.500	5,435	0.483	0.500

Abbreviations: N: sample size; SD: standard deviation; Unmatched data: full set of data; Matched data: subset of data which excluded outliers in observed variables.

**Table 8:** Descriptive statistics for variables used in analyzing medical care utilization of under-five children who reported ARI/diarrhea/fever in the prior two weeks of the survey (pooled RDHS 2005 and 2008).

	Unmatched Data			Matched Data		
	N	Mean	SD	N	Mean	SD
*Dependent Variables*						
Childcare	4,633	0.287	0.453	4,421	0.290	0.454
*Independent Variables*						
*Mutuelles* coverage	4,633	0.501	0.500	4,421	0.500	0.500
Head: age	4,633	37.10	11.061	4,421	36.70	10.89
Head: female	4,633	0.179	0.384	4,421	0.169	0.375
Mother: age	4,633	30.45	6.774	4,421	30.19	6.65
Mother's schooling	4,633	0.728	0.445	4,421	0.737	0.440
Rural residence	4,633	0.791	0.407	4,421	0.795	0.404
1^st^ wealth quintile	4,633	0.191	0.393	4,421	0.185	0.388
2^nd^ wealth quintile	4,633	0.226	0.418	4,421	0.225	0.418
3^rd^ wealth quintile	4,633	0.192	0.394	4,421	0.194	0.396
4^th^ wealth quintile	4,633	0.202	0.402	4,421	0.204	0.403
5^th^ wealth quintile	4,633	0.189	0.392	4,421	0.192	0.393
Radio ownership	4,596	0.517	0.500	4,421	0.568	0.743

Abbreviations: N: sample size; SD: standard deviation; Unmatched data: full set of data; Matched data: subset of data which excluded outliers in observed variables.

**Table 9:** Descriptive statistics for variables used in analyzing utilization of skilled-birth attendance (pooled RDHS 2005 and 2008).

	Unmatched Data			Matched Data		
	N	Mean	SD	N	Mean	SD
*Dependant Variables*						
Skilled birth attendance	1,855	0.601	0.490	1,766	0.601	0.490
*Independent Variables*						
*Mutuelles* coverage	1,832	0.561	0.496	1,766	0.560	0.560
Head: age	1,855	35.64	10.934	1,766	35.14	10.55
Head: female	1,855	0.151	0.359	1,766	0.149	0.356
Woman's age	1,838	28.92	6.43	1,766	28.83	6.34
Woman's schooling	1,855	0.765	0.424	1,766	0.767	0.422
Rural residence	1,855	0.795	0.404	1,766	0.797	0.402
1^st^ wealth quintile	1,855	0.183	0.387	1,766	0.184	0.388
2^nd^ wealth quintile	1,855	0.236	0.424	1,766	0.232	0.422
3^rd^ wealth quintile	1,855	0.199	0.400	1,766	0.203	0.403
4^th^ wealth quintile	1,855	0.194	0.396	1,766	0.201	0.400
5^th^ wealth quintile	1,855	0.188	0.390	1,766	0.180	0.384
Radio ownership	1,852	0.522	0.500	1,766	0.516	0.500

Abbreviations: N: sample size; SD: standard deviation; Unmatched data: full set of data; Matched data: subset of data which excluded outliers in observed variables.

**Table 10:** Descriptive statistics for variables used in analyzing household catastrophic health spending (EICV 2006).

	Unmatched Data			Matched Data		
	N	Mean	SD	N	Mean	SD
***Dependent Variables***						
Catastrophic health spending	6,264	0.080	0.271	5,432	0.082	0.274
***Independent Variables***						
Head: *Mutuelles* coverage	6,263	0.401	0.490	5,446	0.400	0.490
Head: age	6,280	44.18	15.43	5,446	44.08	15.39
Head: female	6,280	0.285	0.451	5,446	0.275	0.447
Head schooling: no schooling	6,280	0.324	0.468	5,446	0.324	0.468
Head schooling: < = primary school	6,280	0.383	0.486	5,446	0.393	0.489
Head schooling: >primary school	6,280	0.293	0.455	5,446	0.283	0.451
Rural residence	6,280	0.797	0.402	5,446	0.824	0.381
Household size	6,280	2.385	0.647	5,446	2.383	0.613
IV expenditure quintile1	6,277	0.203	0.402	5,443	0.193	0.395
IV expenditure quintile2	6,277	0.197	0.398	5,443	0.209	0.407
IV expenditure quintile3	6,238	0.202	0.402	5,443	0.217	0.412
IV expenditure quintile4	6,277	0.207	0.405	5,443	0.210	0.407
IV expenditure quintile5	6,277	0.191	0.393	5,443	0.171	0.377
Under-five children	6,280	0.598	0.490	5,446	0.602	0.490
Elderly (≥60)	6,280	0.163	0.370	5,446	0.162	0.368
Disability	6,280	0.164	0.370	5,446	0.161	0.367
Time to health center (>1 hour)	6,280	0.365	0.481	5,446	0.367	0.482
Time to hospital (>2 hours)	6,276	0.609	0.488	5,446	0.620	0.485

Abbreviations: N: sample size; SD: standard deviation; Unmatched data: full set of data; Matched data: subset of data which excluded outliers in observed variables.

In the unmatched data of EICV 2006 that includes 6,334 individuals who reported an illness in the previous two weeks, about 36 percent of them were *Mutuelles* enrollees. 30 percent of the sampled population used medical care. In the matched dataset with 5,435 individuals who reported illnesses, the coverage of *Mutuelles* is about the same as that of the unmatched data.

About 31 percent of the matched data used medical care (Table 7).

Table 8 shows that, with the pooled data (including unmatched and matched) from the RDHS 2005 and 2008, about 50 percent of the under-five children were *Mutuelles* enrollees.

About 29 percent of sick children received medical care. Table 9 shows, with pooled data from the RDHS 2005 and 2008, about 56 percent of women who had deliveries were *Mutuelles* enrollees. About 60 percent of the deliveries had skilled birth attendance.

With household data (both unmatched and matched) from EICV 2006, Table 10 shows 40 percent of households were covered by *Mutuelles* and about eight percent of total households had catastrophic health spending. Table 11 presents summary statistics for aggregated variables used in the panel data analyses at the provincial level.

**Table 11:** Descriptive statistics of provincial-level variables.

	N	Mean	SD
**Child care utilization analysis**			
*Dependent Variables*			
% of under-five children obtained care when in sick	30	0.230	0.111
*Independent Variables*			
% of children enrolled in *Mutuelles*	30	0.361	0.287
% of mother obtained some schooling	30	0.696	0.089
% of children living in the poorest quintile	30	0.188	0.069
**Skilled-birth attendance utilization analysis**			
*Dependent Variables*			
% of women with skilled-birth attendance in their delivery	30	0.502	0.172
*Independent Variables*			
% of women with delivery enrolled in *Mutuelles*	30	0.368	0.299
% of women with delivery obtained some schooling	30	0.712	0.087
% of women with delivery living in the poorest quintile	30	0.181	0.067

Abbreviations: N: sample size; SD: standard deviation.

### Impact of Mutuelles on medical care utilization

**Figure 1:**
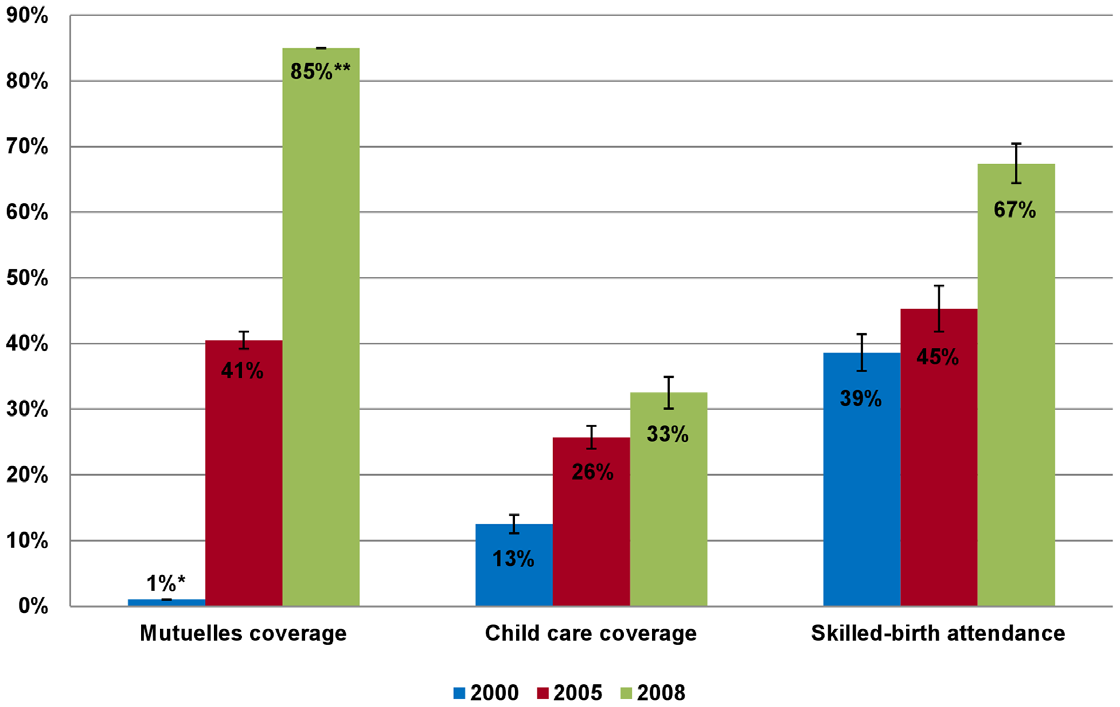
Trends of *Mutuelles* coverage and utilization of child care and skilled-birth attendance. The trends are between 2000 and 2008. The data is taken from the Rwanda Demographic and Health Survey in 2000, 2005, and 2008. Error bars represent 95% confidence intervals (CI). * Estimate is based on a study by Schneider and Diop in 2004 [20]. ** Estimate is from Community Based Health Insurance in Rwanda (http://www.cbhirwanda.org.rw/) [16].

At the national level, the percentage of the population covered by *Mutuelles* rose from about one percent in 2000 to 85 percent in 2008. During the same period, medical care utilization for under-five children with ARI, diarrhea, or fever increased from 13 percent in 2000 to 33 percent in 2008, and the utilization of skilled-birth attendants rose from 39 percent in 2000 to 67 percent in 2008 (Figure 1). Table 12 shows that among under-five children who reported having ARI, diarrhea, or fever, and women who had a delivery, *Mutuelles* enrollees reported significantly higher rates of medical care utilization than the uninsured in the survey year. The difference between years was statistically significant. Between 2000 and 2008, under-five child mortality, infant mortality, and maternal mortality also declined drastically and are lower than the average estimates in the sub-Saharan countries (Table 13).

**Table 12:** Self-reported medical care utilization when ill by *Mutuelles* status.

	Use of medical care
	Mean (95% CI)
**Under-five children 2005 (RDHS 2005)**	
No insurance	0.206 (0.186, 0.227)
With *Mutuelles*	0.327 (0.299, 0.355)
**Under-five children 2008 (RDHS 2008)**	
No insurance	0.208 (0.175, 0.240)
With *Mutuelles*	0.393 (0.362, 0.423)

	Use of skilled-birth attendance
**Women 2005 (RDHS 2005)**	
No insurance	0.379 (0.330, 0.428)
With *Mutuelles*	0.535 (0.478, 0.592)
**Women 2008 (RDHS 2008)**	
No insurance	0.595 (0.542, 0.648)
With *Mutuelles*	0.719 (0.683, 0.756)

**Table 13:** Improved health outcome indicators over time.

	2000	2005	2008	2010
Under-five mortality rate (per 1,000 live births)	196	152	103(133: sub-Saharan area)	76
Infant mortality rate (per 1,000 live births)	107	86	62(83: sub-Saharan area)	50
Maternal mortality ratio (per 100,000 live births)	1,071	750	540(640: sub-Saharan are)	NA

Source: WHO, UNICEF, UNFPA and the World Bank (http://www.childinfo.org/maternal_mortality.html).

Table 14 shows that at the provincial level, *Mutuelles* coverage had a positive and significant effect on child care and maternal care coverage after adjusting for possible confounders such as the percentage of population in the lowest wealth quintile, mothers' or women's schooling level, and the time-invariant unobserved characteristics of the provinces.

**Table 14:** Regression results for child and maternal care analyses with panel data at the provincial level.

Child care coverage analysis with random-effects model	Coefficient	SE	P Value
% of population enrolled in *Mutuelles*	0.327	0.045	0.000**
% of mother with some schooling	0.139	0.117	0.236
% of population in the lowest wealth quintile	−0.278	0.115	0.015*
Constant	0.067	0.084	0.422
*N = 30, R^2^ = 0.774*			
**Skilled-birth attendance analysis with random-effects model**			
% of population enrolled in *Mutuelles*	0.407	0.090	0.000**
% of women with some schooling	0.419	0.169	0.013*
% of population in the lowest wealth quintile	−0.774	0.312	0.013*
Constant	0.194	0.100	0.052
*N = 30, R^2^ = 0.643*			

Abbreviations: SE: standard error; N: sample size.

* : statistically significant at the 0.05 level.

** : statistically significant at the 0.01 level.

Table 15 presents the logistic regression results generated from the unmatched data, matched data, and matched data with IV method for utilization analysis among the general population that reported illnesses in the two weeks prior to the survey. The findings on *Mutuelles* are consistent across the three datasets: *Mutuelles* enrollees were more likely to use medical services than those without any insurance after controlling for other factors. The odds of using medical care increased by 2 for *Mutuelles* enrollees.

**Table 15:** Logistic regression results for medical care utilization among the general population who reported illness using unmatched data, matched data, and matched data with IV method.

	Unmatched Data			Matched Data			Matched Data with IV		
	(N = 6,317)			(N = 5,435)			(N = 5,331)		
Medical care utilization	OR	SE	P Value	OR	SE	P Value	OR	SE	P Value
*Mutuelles* coverage	2.164	0.136	0.000**	2.124	0.140	0.000**	1.886	0.494	0.015*
Age <5 (reference)	1.000	---	---	1.000	---	---	1.000	---	---
Age 5–18	0.468	0.041	0.000**	0.482	0.044	0.000**	0.501	0.046	0.000**
Age 18–30	0.639	0.057	0.000**	0.664	0.062	0.000**	0.674	0.064	0.000**
Age 30–45	0.571	0.056	0.000**	0.587	0.061	0.000**	0.593	0.061	0.000**
Age 45–60	0.463	0.052	0.000**	0.474	0.058	0.000**	0.501	0.061	0.000**
Age >60	0.437	0.059	0.000**	0.447	0.067	0.000**	0.464	0.069	0.000**
Female	0.913	0.056	0.228	0.919	0.058	0.185	0.911	0.058	0.142
Head: no schooling (reference)	1.000	---	---	1.000	---	---	1.000	---	---
Head: < = primary schooling	1.044	0.078	0.560	1.049	0.083	0.541	1.035	0.082	0.664
Head:: >primary schooling	1.146	0.094	0.096	1.101	0.099	0.282	1.080	0.099	0.399
Rural residence	1.036	0.119	0.761	0.933	0.122	0.597	0.836	0.114	0.191
Household Size	1.187	0.060	0.001**	1.170	0.066	0.005**	1.127	0.065	0.038*
Expenditure quintile1	0.394	0.044	0.000**	0.457	0.057	0.000**	0.489	0.064	0.000**
Expenditure quintile2	0.604	0.060	0.000**	0.692	0.075	0.001**	0.730	0.082	0.005**
Expenditure quintile3	0.633	0.060	0.000**	0.726	0.074	0.002**	0.745	0.076	0.004**
Expenditure quintile4	0.709	0.065	0.001**	0.784	0.078	0.015*	0.799	0.080	0.024**
Expenditure quintile5 (reference)	1.000	---	---	1.000	---	---	1.000	---	---
Severity of illness	2.948	0.220	0.000**	3.096	0.251	0.000**	3.007	0.243	0.000**
Disability	0.886	0.115	0.351	0.831	0.119	0.197	0.812	0.119	0.153
Distance to health center (>1 hour)	0.814	0.053	0.002**	0.814	0.058	0.004**	0.840	0.060	0.014*
Distance to hospital (>2 hours)	0.921	0.066	0.250	0.966	0.075	0.660	0.984	0.077	0.835
Radio ownership	1.164	0.074	0.017*	1.139	0.075	0.049*	1.163	0.082	0.033*
Regional dummies (omitted)									

Abbreviations: N: sample size, OR: odds ratio, SE: standard error.

* : statistically significant at the 0.05 level.

** : statistically significant at the 0.01 level.

In addition to *Mutuelles* coverage, significant predictors of medical care utilization included self-reported severity of illness, age, household size, expenditure quintiles, travel time to health center, and radio ownership. For example, those who reported severe illnesses were about three times more likely to use care. Compared to individuals in the highest expenditure quintile, individuals in other quintiles were less likely to use care. Individuals with shorter travel time to the nearest health facilities were more likely to use care.

**Figure 2:**
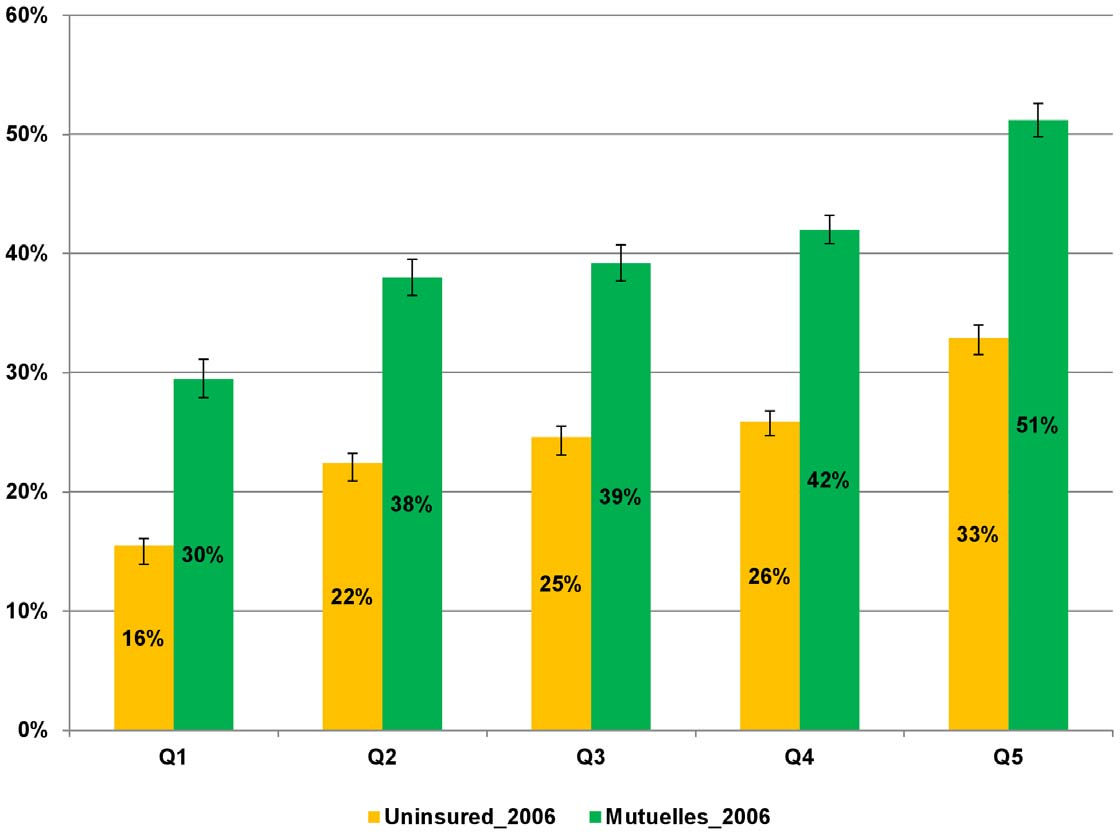
Probability of using medical care when ill by expenditure quintiles, controlling for observable confounders. The numbers are generated from a regression analysis of medical care utilization among the general population using the Integrated Living Conditions Survey (EICV) 2006. Error bars represent 95% confidence intervals (CI).

Figure 2 presents the likelihood of using care for *Mutuelles* enrollees and the uninsured population across expenditure quintiles after controlling for possible confounders using EICV 2006. In each expenditure quintile, the probability of using care among *Mutuelles* enrollees was significantly higher than that among uninsured individuals. Among *Mutuelles* enrollees, those in the lowest expenditure quintile had the lowest probability of using care when ill.

Using pooled data from the RDHS in 2005 and 2008, Table 16 shows that among under-five children that reported diarrhea, fever, or ARI in the two weeks prior to the survey, *Mutuelles* enrollees were more likely to use medical care. The results of *Mutuelles* impact are consistent across unmatched data, matched data, and matched data with IV methods. Other significant predictors included “Year 2008” (2005 as reference), wealth quintiles, and radio ownership. For example, holding everything else constant, children in 2008 were more likely to obtain care than children in 2005 when they were ill.

**Table 16:** Logistic regression results for child care utilization using unmatched data, matched data, and matched data with IV method.

	Unmatched Data (N = 4,596)			Matched Data (N = 4,421)			Matched data with IV (N = 4,203)		
Child care utilization	OR	SE	P Value	OR	SE	P Value	OR	SE	P Value
*Mutuelles* coverage	2.010	0.154	0.000**	2.002	0.154	0.000**	3.398	0.901	0.000**
Year 2005 (reference)	1.000	---	---	1.000	---	---	1.000	---	---
Year 2008	1.297	0.117	0.004**	1.309	0.119	0.003**	1.171	0.126	0.144*
Head: age <30 (reference)	1.000	---	---	1.000	---	---	1.000	---	---
Head: age 30–50	0.990	0.097	0.915	0.980	0.097	0.839	0.961	0.097	0.696
Head: age >50	0.942	0.125	0.654	0.951	0.127	0.707	0.934	0.127	0.619
Head: female	1.016	0.100	0.872	1.025	0.102	0.806	0.992	0.103	0.936
Mother's age	0.915	0.080	0.311	0.923	0.083	0.371	0.936	0.085	0.470
Mother's schooling	1.025	0.091	0.779	1.017	0.091	0.855	1.031	0.098	0.752
Rural residence	0.861	0.088	0.140	0.846	0.087	0.105	0.834	0.090	0.091
Wealth quintile1	0.529	0.077	0.000**	0.531	0.078	0.000**	0.572	0.087	0.000**
Wealth quintile2	0.452	0.057	0.000**	0.456	0.058	0.000**	0.447	0.059	0.000**
Wealth quintile3	0.465	0.056	0.000**	0.466	0.057	0.000**	0.458	0.058	0.000**
Wealth quintile4	0.552	0.067	0.000**	0.559	0.068	0.000**	0.556	0.070	0.000**
Wealth quintile5 (reference)	1.000	---	---	1.000	---	---	1.000	---	---
Radio ownership	1.315	0.123	0.003**	1.310	0.123	0.004**	1.383	0.132	0.001**

Abbreviations: N: sample size, OR: odds ratio, SE: standard error.

* : statistically significant at the 0.05 level.

** : statistically significant at the 0.01 level.

Table 17 shows that among women who delivered children in the survey years, *Mutuelles* enrollees were more likely to use skilled-birth attendance. The results of *Mutuelles* impact are consistent across the unmatched data, matched data, and matched data with IV methods. Other significant predictors included wealth quintiles, women's age and schooling level.

**Table 17:** Logistic regression results for skilled-birth attendance utilization using unmatched data, matched data, and matched data with IV method.

	Unmatched Data (N = 1,852)			Matched data (N = 1,766)			Matched data with IV (N = 1,700)		
Skilled-birth attendance	OR	SE	P Value	OR	SE	P Value	OR	SE	P Value
*Mutuelles* coverage	1.778	0.203	0.000**	1.779	0.206	0.000**	2.630	1.124	0.024*
Year 2005 (reference)	1.000	---	---	1.000	---	---	1.000	---	---
Year 2008	2.304	0.304	0.000**	2.338	0.314	0.000**	2.097	0.330	0.000**
Head: age <30 (reference)	1.000	---	---	1.000	---	---	1.000	---	---
Head: age 30–50	0.871	0.122	0.323	0.859	0.122	0.285	0.848	0.123	0.257
Head: age >50	0.826	0.174	0.364	0.825	0.184	0.389	0.760	0.167	0.213
Head: female	1.127	0.182	0.459	1.083	0.180	0.630	1.095	0.184	0.590
Woman's age	0.472	0.059	0.000**	0.477	0.061	0.000**	0.477	0.061	0.000**
Woman's schooling	1.953	0.233	0.000**	2.041	0.254	0.000**	1.895	0.240	0.000**
Rural residence	0.634	0.114	0.011*	0.662	0.123	0.026*	0.703	0.132	0.061
Wealth quintile1	0.366	0.075	0.000**	0.369	0.078	0.000**	0.367	0.079	0.000**
Wealth quintile2	0.433	0.084	0.000**	0.442	0.088	0.000**	0.423	0.085	0.000**
Wealth quintile3	0.447	0.087	0.000**	0.463	0.092	0.000**	0.434	0.087	0.000**
Wealth quintile4	0.574	0.113	0.005**	0.589	0.118	0.008**	0.568	0.116	0.005**
Wealth quintile5 (reference)	1.000	---	---	1.000	---	---	1.000	---	---
Radio ownership	1.081	0.125	0.501	1.067	0.126	0.580	1.049	0.125	0.686

Abbreviations: N: sample size, OR: odds ratio, SE: standard error.

* : statistically significant at the 0.05 level.

** : statistically significant at the 0.01 level.

### Impact of Mutuelles on household financial risk protection

**Figure 3:**
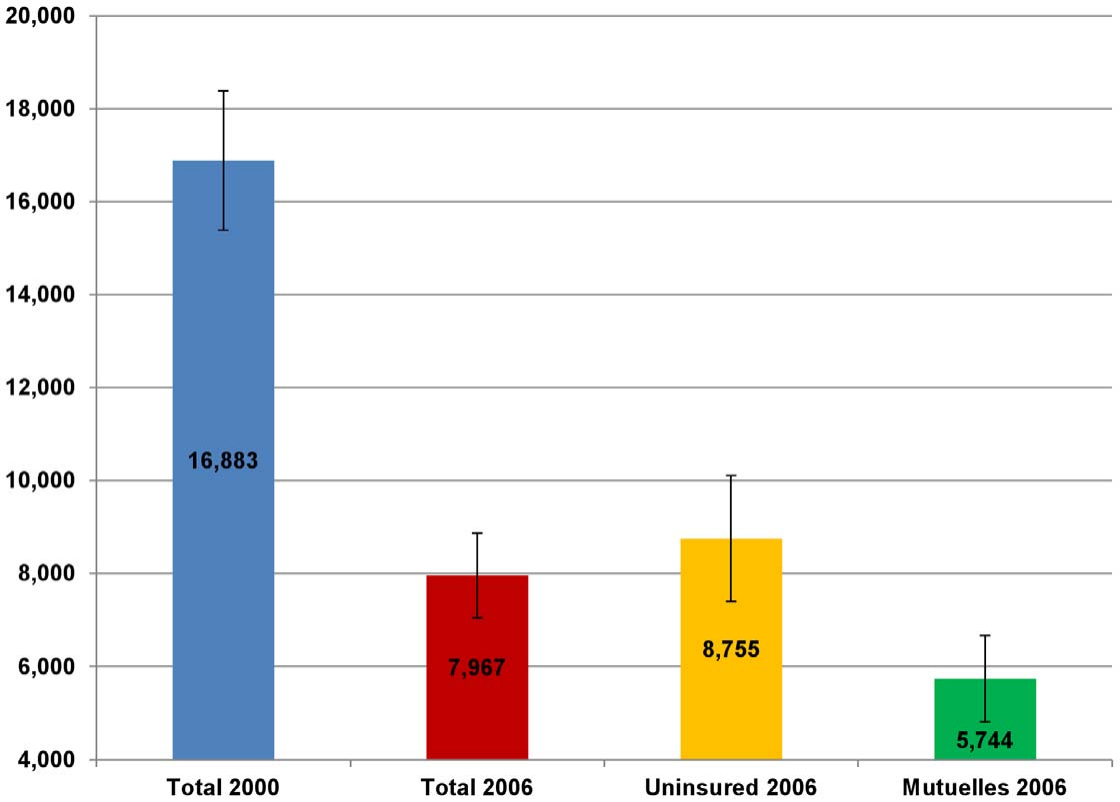
Average annual household out-of-pocket health spending (in 2000 RWF) in 2000 and 2006. The data is taken from the Integrated Living Conditions Survey (EICV) 2000 and 2006. Error bars represent 95% confidence intervals (CI).

**Figure 4:**
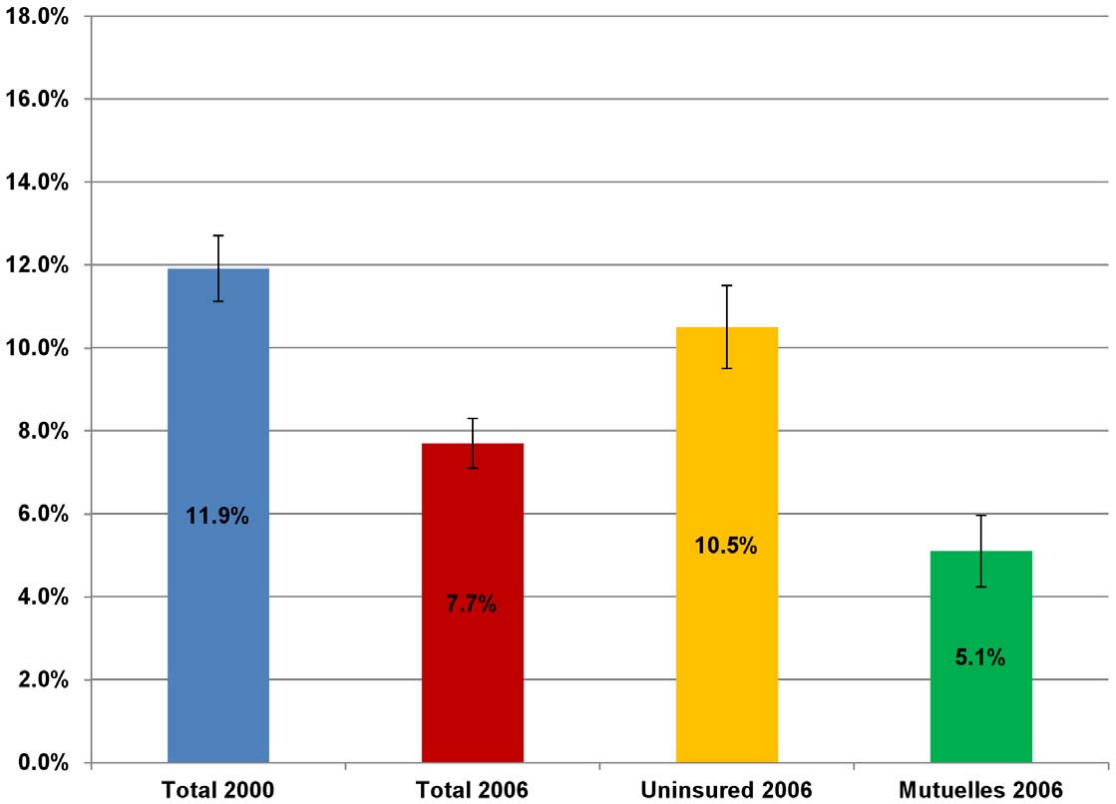
Percentage of Rwanda households with catastrophic health spending in 2000 and 2006. The data is taken from the Integrated Living Conditions Survey (EICV) 2000 and 2006. Error bars represent 95% confidence intervals (CI).

Between 2000 and 2006, the average Rwandan annual household OOPS (in 2000 RWF) fell significantly from 16,883 to 7,967 RWF (in 2000 RWF) (Figure 3), while the percentage of households incurring catastrophic health spending fell significantly from 11.9 percent to 7.7 percent (Figure 4). In 2006, the average annual household OOPS for *Mutuelles* holders (5,744 RWF) was significantly lower than that of the uninsured households (8,755 RWF) (Figure 3). The percentage of the *Mutuelles* households with catastrophic spending (5.1 percent) was significantly lower than that (10.5 percent) of uninsured households (Figure 4).

Table 18 shows that after controlling for possible confounders, *Mutuelles* households were less likely to incur catastrophic health spending. Other significant predictors of catastrophic health spending included the presence of disabled household members and under-five children, instrumented expenditure quintiles, and household size. For example, households in the lowest expenditure quintile were about 8 to 10 times more likely to incur catastrophic health spending. The results are robust to various datasets and estimation methods.

**Table 18:** Regression results of household catastrophic health spending with unmatched data, matched data, and matched data with IV method (EICV 2006).

	Unmatched Data (N = 6,241)			Matched Data (N = 5,430)			Matched Data with IV (N = 5,430)		
	OR	SE	P Value	OR	SE	P Value	OR	SE	P Value
*Mutuelles* coverage	0.654	0.075	0.000**	0.682	0.080	0.001**	0.047	0.020	0.000**
Head: age<30 (reference group)	1.000	---	---	1.000	---	---	1.000	---	---
Head: age 30–50	0.827	0.107	0.143	0.873	0.123	0.333	0.905	0.127	0.475
Head: age >50	0.780	0.182	0.287	0.863	0.210	0.545	0.980	0.239	0.934
Head: female	0.806	0.103	0.091	0.782	0.110	0.079	0.711	0.100	0.015*
Head: no schooling (reference group)	1.000	---	---	1.000	---	---	1.000	---	---
Head: < = primary school	0.965	0.112	0.762	0.991	0.124	0.945	1.046	0.133	0.725
Head: >primary school	1.057	0.171	0.732	1.260	0.216	0.179	1.620	0.283	0.006**
Rural residence	0.904	0.162	0.574	1.142	0.277	0.583	1.318	0.326	0.264
Household size	1.664	0.238	0.000**	1.811	0.284	0.000**	2.023	0.319	0.000**
IV expenditure quintile1	10.670	3.476	0.000**	8.763	3.015	0.000**	7.556	2.592	0.000**
IV expenditure quintile2	7.011	2.055	0.000**	5.483	1.710	0.000**	4.945	1.537	0.000**
IV expenditure quintile3	4.469	1.189	0.000**	3.414	0.976	0.000**	3.161	0.908	0.000**
IV expenditure quintile4	1.941	0.506	0.011*	1.478	0.415	0.163	1.449	0.406	0.185
IV expenditure quintile5 (reference group)	1.000	---	---	1.000	---	---	1.000	---	---
Under-five children	1.832	0.267	0.000**	1.799	0.276	0.000**	1.708	0.263	0.001**
Elderly (≥60)	1.036	0.240	0.878	0.992	0.241	0.973	0.964	0.238	0.881
Disability	1.418	0.179	0.006**	1.504	0.200	0.002**	1.491	0.200	0.003**
Distance to health center (>1 hour)	1.141	0.116	0.194	1.104	0.119	0.357	1.013	0.111	0.903
Distance to hospital (>2 hours)	1.038	0.122	0.748	1.068	0.133	0.595	1.079	0.136	0.547
Regional dummies (omitted)									

Abbreviations: N: sample size, OR: odds ratio, SE: standard error.

* : statistically significant at the 0.05 level.

** : statistically significant at the 0.01 level.

**Figure 5:**
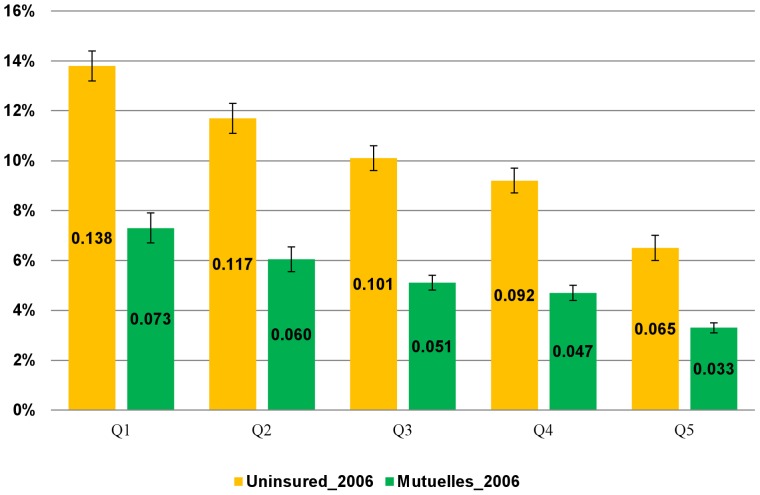
Probability of incurring catastrophic health spending by expenditure quintiles, controlling for observable confounders. The numbers are generated from a regression analysis of household incurring catastrophic health spending, using the Integrated Living Conditions Survey (EICV) 2006. Error bars represent 95% confidence intervals (CI).

Figure 5 shows that in each expenditure quintile, *Mutuelles* households had a significantly lower probability of incurring catastrophic health spending than the uninsured households after controlling for observed confounding factors using EICV 2006. Among *Mutuelles* households, households in the lowest quintile had the highest probability of experiencing catastrophic health spending.

## Discussion

This study utilizes the available nationally-representative surveys in Rwanda and traces the evolution of *Mutuelles* coverage and its relationship with child and maternal care from 2000 to 2008, as well as household catastrophic health payments from 2000 to 2006. Using statistical models, it examines the two most important expected results of *Mutuelles* at the individual and household level: an increase in using care and a reduction in the incidence of catastrophic health spending. The evidence suggests that at the individual level, *Mutuelles* improved medical care utilization among the general population, under-five children, and women with child delivery. At the household level, *Mutuelles* protected households from catastrophic health spending. At the provincial level, we found a positive effect of *Mutuelles* coverage on child and maternal care coverage. These findings are robust to various estimation methods and datasets. At the national level, we observed an increase in medical care coverage accompanied by a decrease in OOPS and percentage of households with catastrophic health spending. It seems plausible that the increase in medical care coverage contributed to major improvements in child mortality and maternal mortality during the same period. Currently, Rwanda is one of the few African countries that stand a chance of reaching the targets of health MDGs [31].

Even with this impressive progress, there is still room for improvement. The utilization rate for curative care is still low for under-five children with acute illnesses (33 percent in 2008). In addition, like many other developing countries, one of the major challenges faced by the GoR is how to ensure that the poorest benefit equally from *Mutuelles*. Findings presented in this paper demonstrate that although *Mutuelles* coverage has substantially increased service utilization and reduced the risk of catastrophic health spending for *Mutuelles* enrollees, *Mutuelles* enrollees in the poorest quintile still had significantly lower rates of utilization and higher rates of experiencing catastrophic health spending than *Mutuelles* enrollees in higher quintiles. The *Mutuelles* copayments may have prevented indigent enrollees, who live under the extreme poverty line of $0.32 per day, from seeking needed care, or placed a heavy economic burden on them when care was sought. The GoR proposed a new version of the Rwanda Community Based Health Insurance Policy in 2010. One of its major components is to reduce copayments for the poorest enrollees [32]. Our future study is to investigate how eliminating *Mutuelles* copayments for the poorest will affect the finance of health care providers, improve health care utilization, and reduce catastrophic health spending for the poorest.

The strengths of this study include (1) providing a comprehensive picture of *Mutuelles'* impact on universal health coverage in its first eight-year implementation by taking advantage of all available nationally-representative data, (2) addressing the aforementioned methodological issues, and (3) examining the robustness of the findings using various datasets and methods. One limitation is that the evaluation of *Mutuelles'* impact on medical care utilization focuses on curative care only. In addition, due to insufficient data, we were not able to measure the impact of *Mutuelles* on health outcomes, such as child or maternal mortality.

The Rwanda experience offers valuable lessons to other low-income countries that are in a similarly challenging situation. The fast expansion of the *Mutuelles* program and high rates of enrollment suggests a strong societal consensus in equal opportunity for everybody to access health care with financial protection. The government played a crucial role through increased financial investment in the health sector, successful legislation of the entitlement of basic care for uninsured population, and an intensive nationwide campaign. The positive results of the *Mutuelles* program in promoting medical care utilization and financial risk protection suggests that the community-based health insurance scheme can be an effective tool for achieving universal health coverage, together with other policy instruments [33].

## Supporting Information

Table S1 **T-tests of mean differences in variables from the matched data for general population who reported illnesses (EICV 2006).**
(DOC)

Table S2 **T-tests of mean differences in variables from the matched data for under-five children who reported illness (pooled RDHS 2005 and 2008).**
(DOCX)

Table S3 **T-tests of mean differences in variables from the matched data for skilled-birth attendance (pooled RDHS 2005 and 2008).**
(DOCX)

Table S4 **T-tests of mean differences in variables from the matched data at the household level (EICV 2006).**
(DOCX)
