# Supplementary material for: Towards Universal Health Coverage: An Evaluation of Rwanda Mutuelles in Its First Eight Years
Source: PLoS One. 2012 Jun 18;7(6):e39282. doi: 10.1371/journal.pone.0039282 (PMC3377670; doi:10.1371/journal.pone.0039282)
Supplement: Table S1 — T-tests of mean differences in variables from the matched data for general population who reported illnesses (EICV 2006). (DOC) [file pone.0039282.s001.doc]

**Table S1.** T-tests of mean differences in variables from the matched data for general population who reported illnesses (EICV 2006).

|  |  | **Mean** | |  | ***t*-test** |
| --- | --- | --- | --- | --- | --- |
| **Variable** | **Sample** | **Treated** | **Control** | **% of Reduced Bias** | **P Value** |
| Age: < 5 | Unmatched | 0.226 | 0.227 |  | 0.910 |
|  | Matched | 0.228 | 0.231 | -145.1 | 0.820 |
| Age 5-18 | Unmatched | 0.226 | 0.243 |  | 0.125 |
|  | Matched | 0.232 | 0.237 | 74.2 | 0.745 |
| Age 18-30 | Unmatched | 0.181 | 0.212 |  | 0.003 |
|  | Matched | 0.197 | 0.197 | 99.2 | 0.984 |
| Age 30-45 | Unmatched | 0.155 | 0.147 |  | 0.389 |
|  | Matched | 0.152 | 0.151 | 83.9 | 0.910 |
| Age 45-60 | Unmatched | 0.124 | 0.100 |  | 0.002 |
|  | Matched | 0.112 | 0.107 | 79.1 | 0.605 |
| Age > 60 | Unmatched | 0.089 | 0.072 |  | 0.019 |
|  | Matched | 0.078 | 0.077 | 95.5 | 0.931 |
| Female | Unmatched | 0.556 | 0.564 |  | 0.545 |
|  | Matched | 0.556 | 0.560 | 51.8 | 0.811 |
| Head: no schooling | Unmatched | 0.268 | 0.345 |  | 0.000 |
|  | Matched | 0.295 | 0.298 | 96.9 | 0.869 |
| Head: <=primary school | Unmatched | 0.392 | 0.404 |  | 0.351 |
|  | Matched | 0.413 | 0.415 | 86.3 | 0.917 |
| Head:  > primary school | Unmatched | 0.340 | 0.251 |  | 0.000 |
|  | Matched | 0.291 | 0.288 | 95.5 | 0.780 |
| Rural residence | Unmatched | 0.843 | 0.788 |  | 0.000 |
|  | Matched | 0.826 | 0.817 | 83.1 | 0.447 |
| Household size | Unmatched | 2.640 | 2.550 |  | 0.000 |
|  | Matched | 2.600 | 2.592 | 96.7 | 0.879 |
| Expenditure quintile1 | Unmatched | 0.131 | 0.224 |  | 0.000 |
|  | Matched | 0.149 | 0.148 | 99.5 | 0.968 |
| Expenditure quintile2 | Unmatched | 0.162 | 0.219 |  | 0.000 |
|  | Matched | 0.180 | 0.199 | 68.0 | 0.143 |
| Expenditure quintile3 | Unmatched | 0.213 | 0.191 |  | 0.031 |
|  | Matched | 0.225 | 0.227 | 92.8 | 0.904 |
| Expenditure quintile4 | Unmatched | 0.249 | 0.182 |  | 0.000 |
|  | Matched | 0.232 | 0.215 | 74.7 | 0.205 |
| Expenditure quintile5 | Unmatched | 0.246 | 0.184 |  | 0.000 |
|  | Matched | 0.214 | 0.212 | 95.7 | 0.842 |
| Severity of Illness | Unmatched | 0.708 | 0.730 |  | 0.061 |
|  | Matched | 0.720 | 0.722 | 88.3 | 0.858 |
| Disability | Unmatched | 0.058 | 0.065 |  | 0.328 |
|  | Matched | 0.060 | 0.057 | 60.6 | 0.745 |
| Time to health center (> 1 hour) | Unmatched | 0.360 | 0.399 |  | 0.002 |
|  | Matched | 0.376 | 0.367 | 76.9 | 0.561 |
| Time to hospital (> 2 hours) | Unmatched | 0.647 | 0.630 |  | 0.197 |
|  | Matched | 0.641 | 0.636 | 72.1 | 0.768 |
| Radio ownership | Unmatched | 0.585 | 0.422 |  | 0.000 |
|  | Matched | 0.530 | 0.505 | 84.3 | 0.108 |
