# Supplementary material for: Towards Universal Health Coverage: An Evaluation of Rwanda Mutuelles in Its First Eight Years
Source: PLoS One. 2012 Jun 18;7(6):e39282. doi: 10.1371/journal.pone.0039282 (PMC3377670; doi:10.1371/journal.pone.0039282)
Supplement: Table S2 — T-tests of mean differences in variables from the matched data for under-five children who reported illness (pooled RDHS 2005 and 2008). (DOCX) [file pone.0039282.s002.docx]

**Table S2.** T-tests of mean differences in variables from the matched data for under-five children who reported illness (pooled RDHS 2005 and 2008).

|  |  | **Mean** | |  | **t-test** |  |
| --- | --- | --- | --- | --- | --- | --- |
| **Variable** | **Sample** | **Treated** | **Control** | **% of Reduced Bias** | **P Value** | |
| Rural residence | Unmatched | 0.813 | 0.768 |  | 0.000 |  |
|  | Matched | 0.817 | 0.809 | 81.6 | 0.478 |  |
| Head: age < 30 | Unmatched | 0.290 | 0.276 |  | 0.287 |  |
|  | Matched | 0.290 | 0.280 | 29.8 | 0.457 |  |
| Head: age 30-50 | Unmatched | 0.609 | 0.596 |  | 0.350 |  |
|  | Matched | 0.614 | 0.610 | 75.1 | 0.815 |  |
| Head: age > 50 | Unmatched | 0.100 | 0.128 |  | 0.003 |  |
|  | Matched | 0.096 | 0.110 | 51.9 | 0.140 |  |
| Head: female | Unmatched | 0.145 | 0.205 |  | 0.000 |  |
|  | Matched | 0.141 | 0.154 | 78.6 | 0.226 |  |
| Wealth quintile1 | Unmatched | 0.165 | 0.216 |  | 0.000 |  |
|  | Matched | 0.165 | 0.177 | 76.5 | 0.284 |  |
| Wealth quintile2 | Unmatched | 0.231 | 0.218 |  | 0.284 |  |
|  | Matched | 0.232 | 0.224 | 35.4 | 0.492 |  |
| Wealth quintile3 | Unmatched | 0.194 | 0.193 |  | 0.961 |  |
|  | Matched | 0.194 | 0.202 | -1267 | 0.509 |  |
| Wealth quintile4 | Unmatched | 0.221 | 0.184 |  | 0.002 |  |
|  | Matched | 0.220 | 0.208 | 67.0 | 0.335 |  |
| Wealth quintile5 | Unmatched | 0.189 | 0.188 |  | 0.928 |  |
|  | Matched | 0.189 | 0.190 | 51.8 | 0.966 |  |
| Mother's age | Unmatched | 0.488 | 0.482 |  | 0.671 |  |
|  | Matched | 0.487 | 0.485 | 55.6 | 0.851 |  |
| Mother's schooling | Unmatched | 0.761 | 0.704 |  | 0.000 |  |
|  | Matched | 0.762 | 0.748 | 75.5 | 0.277 |  |
| Radio ownership | Unmatched | 0.601 | 0.436 |  | 0.000 |  |
|  | Matched | 0.603 | 0.575 | 82.9 | 0.057 |  |
