# Supplementary material for: Towards Universal Health Coverage: An Evaluation of Rwanda Mutuelles in Its First Eight Years
Source: PLoS One. 2012 Jun 18;7(6):e39282. doi: 10.1371/journal.pone.0039282 (PMC3377670; doi:10.1371/journal.pone.0039282)
Supplement: Table S3 — T-tests of mean differences in variables from the matched data for skilled-birth attendance (pooled RDHS 2005 and 2008). (DOCX) [file pone.0039282.s003.docx]

**Table S3.** T-tests of mean differences in variables from the matched data for skilled-birth attendance (pooled RDHS 2005 and 2008).

|  |  | **Mean** |  | **t-test** |  |
| --- | --- | --- | --- | --- | --- |
| **Variable** | **Sample** | **Treated** | **Control** | **% of Reduced Bias** | **P Value** |
| Rural residence | Unmatched | 0.811 | 0.776 |  | 0.067 |
|  | Matched | 0.807 | 0.795 | 66.90 | 0.518 |
| Head: age < 30 | Unmatched | 0.323 | 0.348 |  | 0.260 |
|  | Matched | 0.331 | 0.342 | 56.20 | 0.604 |
| Head: age 30-50 | Unmatched | 0.568 | 0.565 |  | 0.890 |
|  | Matched | 0.584 | 0.572 | -268.50 | 0.589 |
| Head: age > 50 | Unmatched | 0.109 | 0.087 |  | 0.122 |
|  | Matched | 0.085 | 0.086 | 95.50 | 0.938 |
| Head: female | Unmatched | 0.149 | 0.158 |  | 0.620 |
|  | Matched | 0.145 | 0.148 | 59.20 | 0.828 |
| Wealth quintile1 | Unmatched | 0.149 | 0.231 |  | 0.000 |
|  | Matched | 0.151 | 0.159 | 89.90 | 0.612 |
| Wealth quintile2 | Unmatched | 0.252 | 0.216 |  | 0.069 |
|  | Matched | 0.243 | 0.241 | 94.50 | 0.917 |
| Wealth quintile3 | Unmatched | 0.204 | 0.194 |  | 0.600 |
|  | Matched | 0.209 | 0.216 | 37.00 | 0.734 |
| Wealth quintile4 | Unmatched | 0.200 | 0.193 |  | 0.700 |
|  | Matched | 0.205 | 0.206 | 92.80 | 0.977 |
| Wealth quintile5 | Unmatched | 0.194 | 0.166 |  | 0.128 |
|  | Matched | 0.192 | 0.179 | 53.30 | 0.458 |
| Women's age | Unmatched | 0.414 | 0.417 |  | 0.889 |
|  | Matched | 0.417 | 0.422 | -61.70 | 0.812 |
| Women's schooling | Unmatched | 0.795 | 0.725 |  | 0.001 |
|  | Matched | 0.792 | 0.782 | 85.50 | 0.583 |
| Radio ownership | Unmatched | 0.529 | 0.504 |  | 0.300 |
|  | Matched | 0.527 | 0.524 | 89.80 | 0.912 |
