# Supplementary material for: Towards Universal Health Coverage: An Evaluation of Rwanda Mutuelles in Its First Eight Years
Source: PLoS One. 2012 Jun 18;7(6):e39282. doi: 10.1371/journal.pone.0039282 (PMC3377670; doi:10.1371/journal.pone.0039282)
Supplement: Table S4 — T-tests of mean differences in variables from the matched data at the household level (EICV 2006). (DOCX) [file pone.0039282.s004.docx]

**Table S4.** T-tests of mean differences in variables from the matched data at the household level (EICV 2006).

|  |  | **Mean** | |  | ***t*-test** |  |
| --- | --- | --- | --- | --- | --- | --- |
| **Variable** | **Sample** | **Treated** | **Control** | **% of Reduced Bias** | **P Value** | |
| Head: age < 30 | Unmatched | 0.315 | 0.350 |  | 0.004 |  |
|  | Matched | 0.336 | 0.335 | 98.4 | 0.969 |  |
| Head: age 30-50 | Unmatched | 0.468 | 0.432 |  | 0.006 |  |
|  | Matched | 0.448 | 0.450 | 95.5 | 0.005 |  |
| Head: age > 50 | Unmatched | 0.217 | 0.218 |  | 0.926 |  |
|  | Matched | 0.216 | 0.215 | -7.1 | 0.932 |  |
| Head: female | Unmatched | 0.239 | 0.315 |  | 0.000 |  |
|  | Matched | 0.259 | 0.267 | 89.8 | 0.561 |  |
| Head: no schooling | Unmatched | 0.287 | 0.350 |  | 0.000 |  |
|  | Matched | 0.311 | 0.320 | 86.0 | 0.530 |  |
| Head: <=primary school | Unmatched | 0.374 | 0.389 |  | 0.227 |  |
|  | Matched | 0.396 | 0.393 | 76.2 | 0.808 |  |
| Head:  > primary school | Unmatched | 0.339 | 0.261 |  | 0.000 |  |
|  | Matched | 0.293 | 0.288 | 93.3 | 0.703 |  |
| Rural residence | Unmatched | 0.851 | 0.762 |  | 0.000 |  |
|  | Matched | 0.838 | 0.835 | 96.5 | 0.784 |  |
| Household size | Unmatched | 2.484 | 2.317 |  | 0.000 |  |
|  | Matched | 2.407 | 2.401 | 97.0 | 0.784 |  |
| IV expenditure Iquintile1 | Unmatched | 0.137 | 0.206 |  | 0.000 |  |
|  | Matched | 0.155 | 0.161 | 91.6 | 0.594 |  |
| IV expenditure quintile2 | Unmatched | 0.162 | 0.203 |  | 0.000 |  |
|  | Matched | 0.179 | 0.195 | 59.6 | 0.163 |  |
| IV expenditure quintile3 | Unmatched | 0.219 | 0.191 |  | 0.006 |  |
|  | Matched | 0.220 | 0.219 | 94.3 | 0.898 |  |
| IV expenditure quintile4 | Unmatched | 0.244 | 0.191 |  | 0.000 |  |
|  | Matched | 0.230 | 0.212 | 65.8 | 0.146 |  |
| IV expenditure quintile5 | Unmatched | 0.238 | 0.209 |  | 0.007 |  |
|  | Matched | 0.217 | 0.214 | 91.4 | 0.841 |  |
| Under-five children | Unmatched | 0.618 | 0.585 |  | 0.009 |  |
|  | Matched | 0.608 | 0.606 | 93.8 | 0.889 |  |
| Elderly (≥ 60) | Unmatched | 0.161 | 0.165 |  | 0.662 |  |
|  | Matched | 0.162 | 0.161 | 77.4 | 0.944 |  |
| Disability | Unmatched | 0.164 | 0.163 |  | 0.926 |  |
|  | Matched | 0.161 | 0.158 | -271.3 | 0.766 |  |
| Time to health center (> 1 hour) | Unmatched | 0.345 | 0.378 |  | 0.008 |  |
|  | Matched | 0.360 | 0.363 | 91.3 | 0.844 |  |
| Time to hospital (> 2 hours) | Unmatched | 0.635 | 0.592 |  | 0.001 |  |
|  | Matched | 0.625 | 0.630 | 87.8 | 0.723 |  |
